# Remodeling the tumor-immune microenvironment by anti-CTLA4 blockade enhanced subsequent anti-PD-1 efficacy in advanced nasopharyngeal carcinoma

**DOI:** 10.1038/s41698-024-00558-1

**Published:** 2024-03-06

**Authors:** Yuxiang Ma, Huaqiang Zhou, Fan Luo, Yang Zhang, Changbin Zhu, Weiwei Li, Zhan Huang, Jingbo Zhao, Jinhui Xue, Yuanyuan Zhao, Wenfeng Fang, Yunpeng Yang, Yan Huang, Li Zhang, Hongyun Zhao

**Affiliations:** 1 Department of Clinical Research, Sun Yat-sen University Cancer Center, State Key Laboratory of Oncology in South China, Guangdong Key Laboratory of Nasopharyngeal Carcinoma Diagnosis and Therapy, Guangdong Provincial Clinical Research Center for Cancer, Guangzhou, China; 2 Department of Medical Oncology, Sun Yat-sen University Cancer Center, State Key Laboratory of Oncology in South China, Guangdong Key Laboratory of Nasopharyngeal Carcinoma Diagnosis and Therapy, Guangdong Provincial Clinical Research Center for Cancer, Guangzhou, China; 3 Intensive Care Unit Department, Sun Yat-sen University Cancer Center, State Key Laboratory of Oncology in South China, Guangdong Key Laboratory of Nasopharyngeal Carcinoma Diagnosis and Therapy, Guangdong Provincial Clinical Research Center for Cancer, Guangzhou, China; 4 Department of Translational Medicine, Amoy Diagnostics Co., Ltd., Xiamen, China; 5 Department of Research and Development, Amoy Diagnostics Co., Ltd., Xiamen, China

**Keywords:** Cancer microenvironment, Head and neck cancer

## Abstract

Sequential immunotherapy has shown certain advantages in malignancy. Here, we aim to evaluate the efficacy of sequential anti-CTLA-4 and anti-PD-1 treatment for recurrent or metastatic nasopharyngeal carcinoma patients (R/M NPC). We retrospectively analysis 2 phase I trial of ipilimumab and camrelizumab in Chinese R/M NPC patients. These patients were initially treated with ipilimumab, a CTLA4 blockade, followed by anti-PD-1 treatment. We observed a durable tumor remission in these patients (mPFS: 12.3 months; mDoR: 20.9 months). Multimodal investigations of biopsy samples disclosed remodeling of tumor-immune microenvironment triggered by ipilimumab. In responders, we found increased tumoral PD-L1/PD-L2 expression and T-cell infiltration after ipilimumab treatment, accompanied by reduced stroma and malignant cell components. In contrast, non-responders exhibited increased B-cell infiltration and increased peripheral CD19 + B cells, suggesting a defective transition from memory B cells to plasma cells. This study proposes that sequential therapy can potentially enhance treatment efficacy in chemotherapy-resistant NPC patients and provides insights into how preexisting anti-CTLA4 blockade can influence subsequent anti-PD-1 efficacy by remodeling the TME. Additionally, our results highlight the need for therapeutic strategies targeting naïve/memory B cells.

## Introduction

Nasopharyngeal carcinoma (NPC) is a common type of malignancy in south China and southeastern Asia^1^. The outcome for patients with recurrent or metastatic (R/M) NPC is poor, with a median overall survival (OS) of about 22 months in the era of chemotherapy^2^. In recent years, the rapid development of immunotherapy has brought a new light to the treatment of NPC. There are several regulatory agency–approved anti-PD-1 agents indicated for the treatment of R/M-NPC (nivolumab, pembrolizumab, camrelizumab, toripalimab, and tislelizumab)^3–8^. Whereas the overall response rate to anti-PD-1/PD-L1 monotherapy in previously treated patients with R/M-NPC is only 20–30%^6,7,9,10^, which is not sufficient to improve clinical outcome in most patients. One effective strategy to improve outcome of immunotherapy is to combine anti-PD-1/PD-L1 with anti-CTLA-4 blockades. However, the recently reported clinical trial of anti-CTLA-4 and anti-PD-L1 combination therapy in R/M NPC demonstrated an unsatisfactory results, with 38% objective response rate (ORR) and 5.9 months median duration of response (mDoR)^11^.

To address this limitation, as displayed by previously trials in melanoma, sequential administration of anti-CTLA4 followed by anti-PD-1 treatment might synergize the effect of these two drugs and make the potential toxicity more controllable^12,13^. In a cohort of 271 patients with melanoma, treatment with sequential anti-CTLA-4 and anti-PD-1 was associated with a better survival outcome than monotherapy with anti-PD-1 or anti-CTLA-4, anti-PD-1 followed by anti-CTLA-4 or dual immunotherapy^12^. Until now, there are no study of sequential immunotherapy in NPC patients. The exploration of its potential application has not been explored to date.

The heterogeneity of tumor-immune microenvironment (TME) results in differences in response to immunotherapy among patients^14^. The TME is generally divided into “immune desert”, “immune excluded” and “inflamed” phenotypes. The inflamed tumor microenvironment is enriched with activated T cells and myeloid cells, and has chemokine, interferon signaling expression. In contrast, in “cold tumors”, that is, immune desert TME, there are only a small number of immune cells or suppressive subpopulations, while effector immune cells cannot effectively infiltrate into the tumor microenvironment and are only distributed in the peripheral stroma^15,16^. Remodeling the tumor-immune microenvironment may further improving immunotherapy for cancer. Previous research indicated that anti-CTLA-4 immunotherapy might re-shape the cellular composition within TME and affect the following anti-PD-1 treatment outcomes^12,17^. For example, all PD-1 blockade responders with metastatic melanoma showed an increase in TCR clonality due to prior CTLA-4 blockade, however, the effect of anti-CTLA-4 on TME has not been further analyzed in this study^18^.

Here, we reported 8 R/M NPC patients who were prior treated with ipilimumab, and subsequently treated with camrelizumab (all the cases were from 2 phase I study: NCT02516527 and NCT02721589)^9,19^. Tumor tissue specimens from 8 patients were selected for RNA sequencing and multiple immunofluorescences. We identified immune subtype-specific signatures associated with prognosis and sought to explore the mechanisms underlying anti-CTLA-4 and anti-PD-1 sequential therapy in patients with NPC.

## Results

### Patient characteristics and clinical efficacy of sequential camrelizumab treatment with prior ipilimumab intervention

**Fig. 1:**
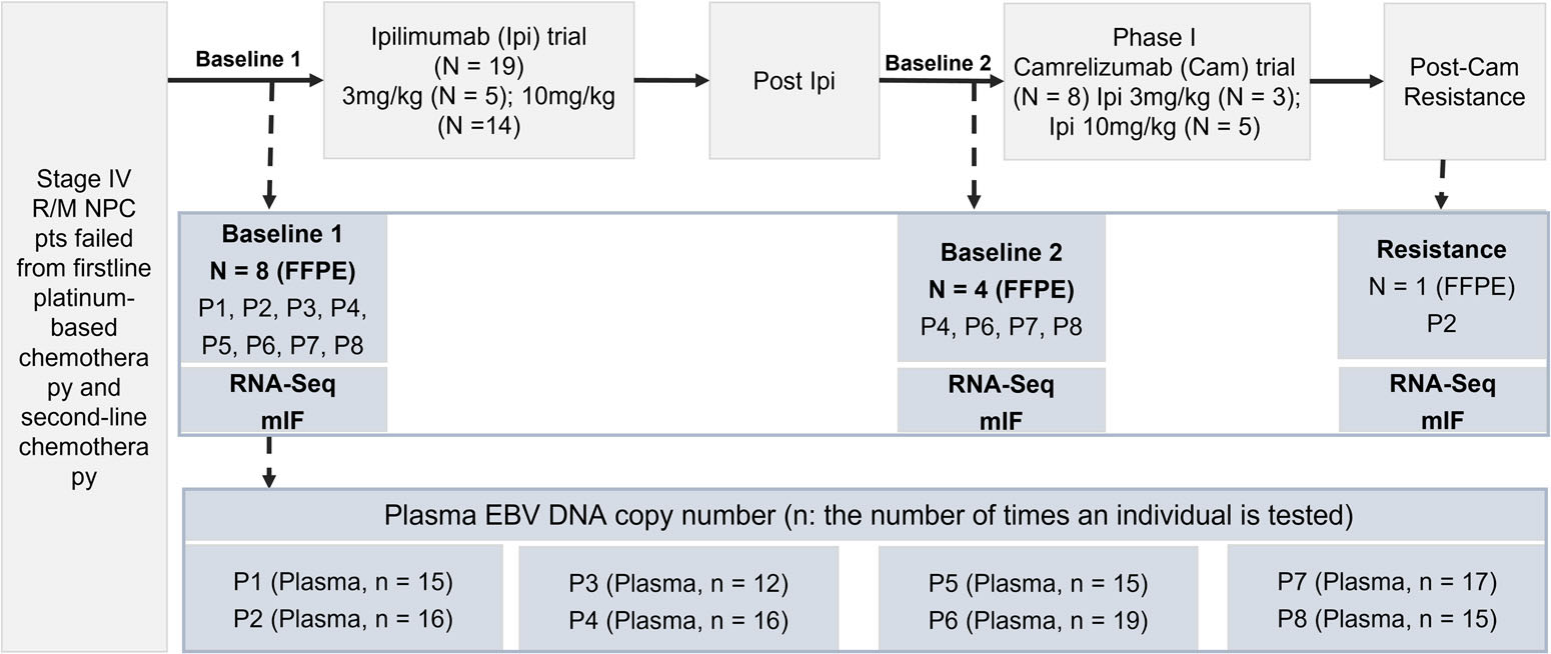
Framework of the study. Flow diagram illustrating the patients included in the analytical process.

**Fig. 2:**
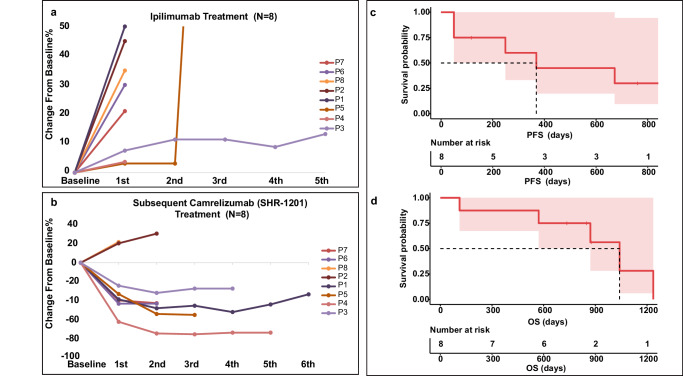
Sequential camrelizumab treatment with prior ipilimumab intervention induced antitumor activation. **a** Spider plot indicating the change in size of the target lesions for each evaluable patient receiving ipilimumab. **b** Spider plot of percentage change in tumor size during sequential camrelizumab treatment. The Kaplan-Meier curves of the PFS **c** and OS **d** for all patients receiving ipilimumab sequential camrelizumab. PFS, progression-free survival; OS, overall survival.

There were 19 patients with advanced NPC receiving Ipilimumab treatment (Fig. 1). In total 8 Ipilimumab pre-treated patients received sequential anti-PD-1 treatment (Fig. 2a). Baseline characteristics were summarized in Supplementary Table 1. Median age was 42 years (range 23–69 years) and most patients are male (*n* = 6, 75%). Liver metastases are found in 6 patients. There were 5 patients with ECOG PS of 0 and 3 with ECOG 1. The average prior treatment line was 3.25. Among these patients, 5 received 10 mg/kg and 3 received 3 mg/kg ipilimumab. Overall, 6 patients who obtained partial response (PR) to camrelizumab were classified as responders, with median DoR of 20.9 months; two patients who showed PD to camrelizumab were classified as non-responders (Fig. 2b). Sequential anti-PD-1 treatment had a median PFS of 12.3 months and median OS of 34.7 months (Fig. 2c, d). Till the last follow-up (2022-11-02), all patients had ceased therapy. The overall incidence of immune-related adverse events (irAEs) was low in 8 patients during camrelizumab treatment, 12.5% (1/8) of patients had grade 3 toxicity. Only 1 patient discontinued treatment due to toxicity and 5 was due to PD.

### Higher PD-L1/PD-L2 expression at baseline or up-regulation after ipilimumab related to sensitivity of subsequent camrelizumab treatment

**Fig. 3:**
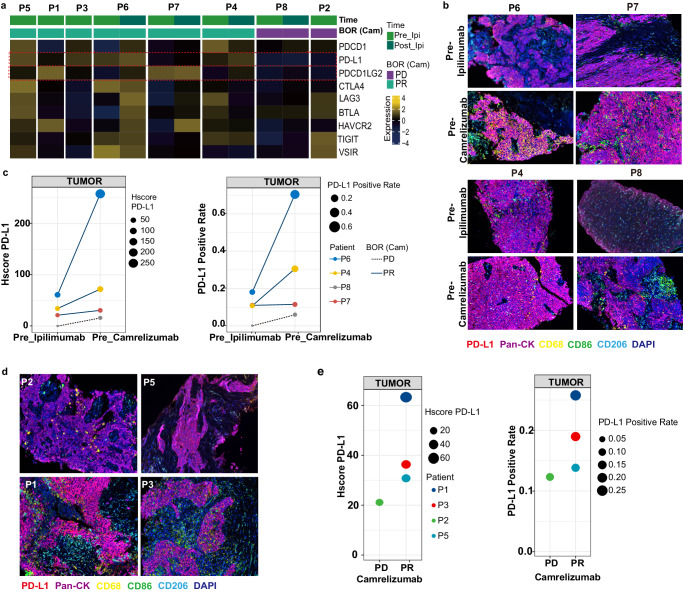
PD-L1 and other immune checkpoint molecular expression in TME. **a** Heatmap showing the expression of immune checkpoint molecules (*PD-L1*, *BTLA*, and *CTLA-4*) and T cell exhaustion genes (*PDCD1*, *LAG3*, *PDCD1LG2*, *HAVCR2*, *TIGIT* and *VSIR*) in representative patients pre-ipilimumab and post-ipilimumab treatment, and in representative patients after camrelizumab treatment. **b** Representative multiplex immunofluorescence image of FFPE samples obtained from patients P6, P7, P4, and P8 pre-and post-ipilimumab treatment. PD-L1 (red), Pan-CK (purple), CD68 (yellow), CD86 (green), CD206 (turquoise), and DAPI (blue). **c** The H-score and positive rate of PD-L1 expression in tumor region pre- and post-ipilimumab treatment of the aforementioned four patients in **b**. **d** Representative multiplex immunofluorescence image of FFPE samples obtained from patients P2, P5, P1, and P3 before ipilimumab treatment or before camralizumab treatment. **e** The H-score and positive rate of PD-L1 expression in tumor region of the aforementioned four patients in **d**.

Higher PD-L1/PD-L2 expression at baseline was found among responders (Fig. 3a, P1, P3, P5). Moreover, expression of PD-L1/PD-L2 was constantly up-regulated due to prior ipilimumab treatment in responders (Fig. 3a, P4, P6, P7). Similarly, mainly NPC cell, but not CD68^+^ CD86^+^ M1 and CD68^+^ CD206^+^ M2 macrophage-derived PD-L1 protein was increased in tumor samples from responders (Fig. 3b and Supplementary Fig. 1). Diminished PD-L1 expression was observed in non-response patients after ipilimumab treatment at transcriptional level (Fig. 3a). Meanwhile, PD-L1 expression, H-score as well as positive rate, was increased in each representative patient after ipilimumab treatment, however the increase in responders was even greater (Fig. 3c). Especially, in P2, upregulation of other immune checkpoints indicated therapeutic opportunities of ICIs might targeting these molecules (Fig. 3a). Multilabel immunofluorescence (mIF) assay were also tested in the other four patients only had tumor tissue sample post-ipilimumab treatment (Fig. 3d and Supplementary Fig. 2). Similarly, PD-L1 expression was higher in patients who responded to camrelizumab than in those who did not (Fig. 3e).

### Ipilimumab-induced remodeling of the TME in camrelizumab-response patients

**Fig. 4:**
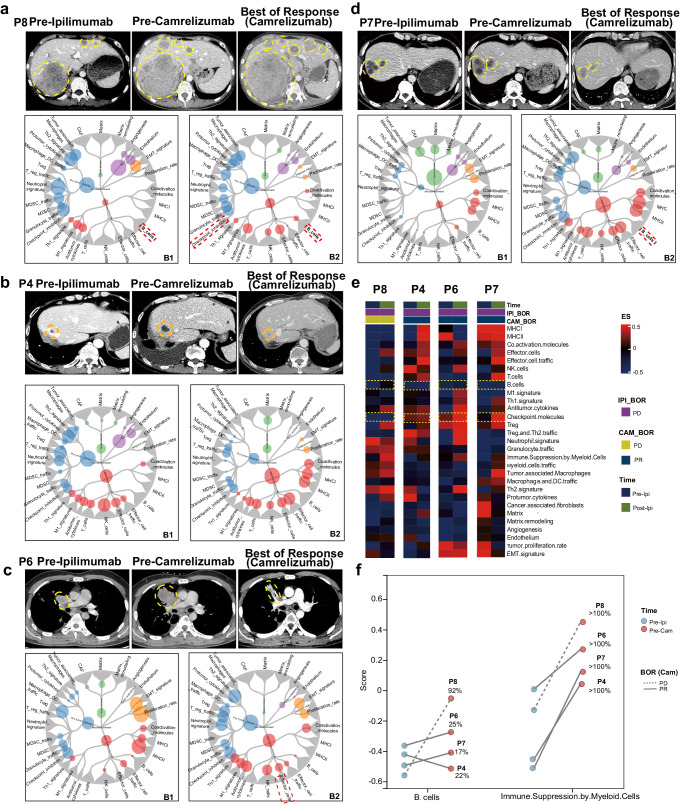
CT images and tumor microenvironment of patients in response and non-response group. **a**–**d** CT images of pre-ipilimumab, pre-camrelizumab, and post-camrelizumab for P8, P4, P6, and P7. Molecular Functional Portrait (potentially targetable genes, signaling pathways, and cellular processes related to each of 29 TME gene expression signatures created by Bagaev et al.^49^) of pre-ipilimumab (B1) and pre-camrelizumab (B2). **e** Heatmap of 29 immuno-related signatures of pre-ipilimumab and pre-camrelizumab for P8, P4, P6, P7. **f** The Enrichment score of B cells and Immune Suppression by Myeloid Cells in tumor region pre-and post-ipilimumab treatment of the aforementioned four patients.

P7 was treated with ipilimumab and sequentially camrelizumab for about 9 months till drop-out due to stomatitis. The corresponding CT demonstrated that his efficacy evaluation of sequential ICIs was PR (Fig. 4d). Anti-tumor immunity signatures, such as anti-tumor cytokines, T cell, and effector cell traffic-related genes, and some pro-tumor immunity signatures, such as MDSC and checkpoint inhibition related genes were enriched after ipilimumab treatment. While tumor stroma, malignant cells, and tumor angiogenesis-related genes were downregulated (Fig. 4d). Similar TME remodeling pattern were observed in P4 (Fig. 4b) and P6 (Fig. 4c). Following camrelizumab treatment, continually reduced plasma EBV DNA copy number was observed (Supplementary Fig. 3), and the decline was related with the best response to camrelizumab (PR).

However, the immune remodeling phenomenon was not obvious in P8 and P2, and their efficacy evaluation of sequential ICIs was PD respectively (Fig. 4a and Supplementary Fig. 4). As for gene expression patterns in TME, the enrichment of the anti-tumor immunity signatures such as anti-tumor cytokines, T cell, effector cell traffic and checkpoint inhibition were not obvious in P8. However, in P8, signatures of the protumor cytokines, malignant cells, and tumor angiogenesis showed the tendency to enrich in tumor microenvironment post-ipilimumab treatment (Fig. 4a, e). As noticed, signatures of myeloid cells (including macrophages, dendritic cells, neutrophils, and myeloid-derived suppressor cells) were prominent to be upregulated in all post-ipilimumab treatment samples regardless of response to camrelizumab (Fig. 4e, f). For P2, due to the lack of specimen before ipilimumab treatment, the changes in TME could not be compared, only the TME analysis results before camrelizumab treatment are shown in Supplementary Fig. 4d. In addition, pre- and post-ICIs treatment plasma EBV DNA copy number was increased during camrelizumab treatment (Supplementary Fig. 3d, e), and the increase was related with poor response to camrelizumab (PD). These data demonstrated that the response to camrelizumab treatment was associated with the TME remodeling of prior ipilimumab treated NPC.

### Intra-tumoral accumulation of CD4^+^ and CD8^+^ T cells after ipilimumab therapy correlated to the efficacy of subsequent treatment of camrelizumab

**Fig. 5:**
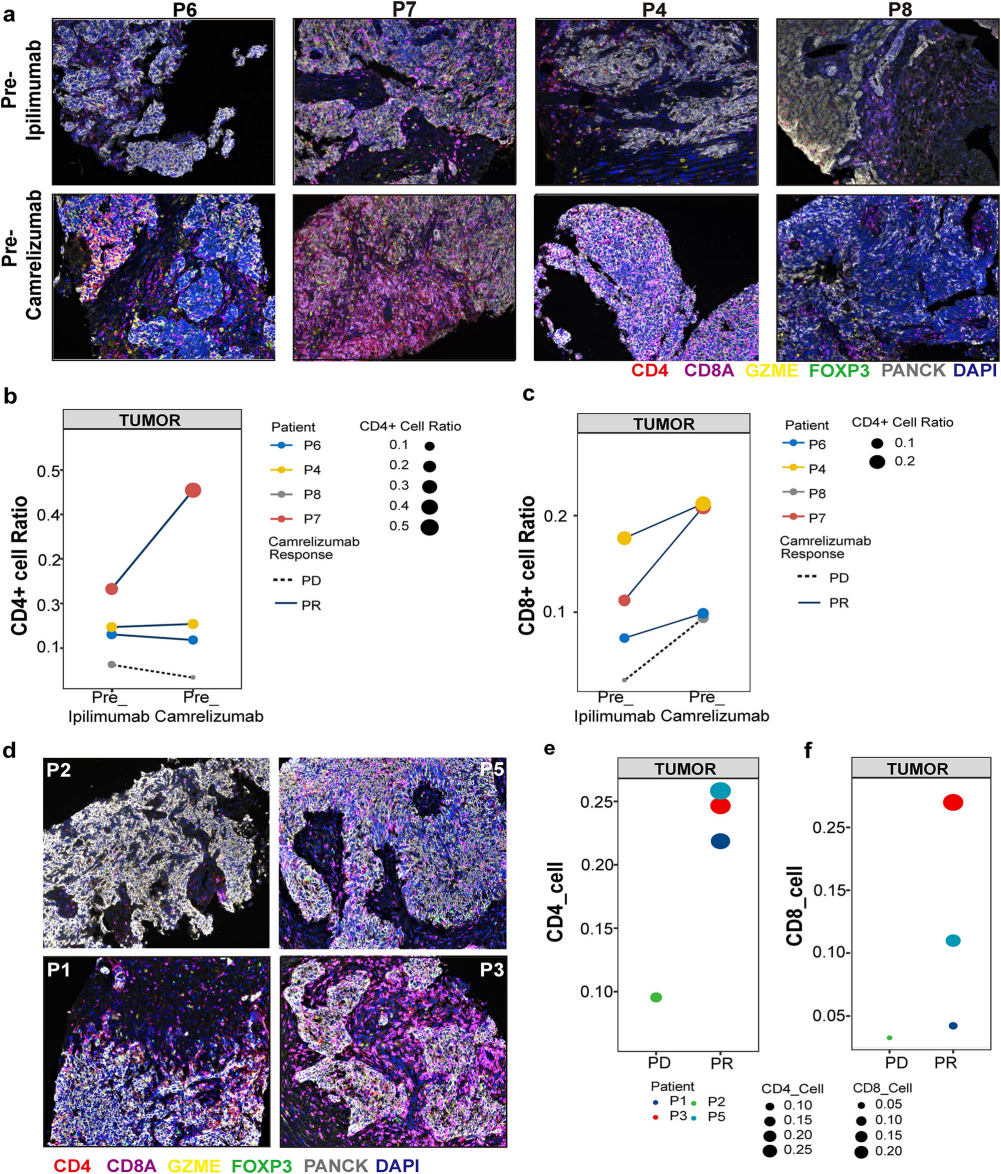
Ipilimumab treatment induced CD4^+^ and CD8^+^ T cells activation and tumor accumulation. **a** Representative multiplex immunofluorescence image of FFPE samples obtained from patients P6, P7, P4, and P8 pre-and post-ipilimumab treatment. CD4 (red), Pan-CD8A (purple), GZME (yellow), FOXP3 (green), Pan-CK (turquoise), and DAPI (blue). The proportion of CD4^+^ **b** and CD8^+^ T cells **c** in tumor of the aforementioned four patients in **a**. **d** Representative multiplex immunofluorescence image of FFPE samples obtained from patients P2, P5, P1, and P3 before ipilimumab treatment or before camrelizumab treatment. The proportion of CD4^+^ **e** and CD8^+^ T cells **f** in tumor of the aforementioned four patients in **d**.

Subsequently, the potential changes in both CD4^+^ and CD8^+^ T effector cell activation in the TME during treatment were examined. mIF on FFPE samples with antibodies of CD4, CD8α, FOXP3, Granzyme B, and pan CK (Fig. 5a and Supplementary Fig. 5) were applied to analyze the tumor regions of pre- and post-ipilimumab treatment for 4 patients (P6, P7, P4 and P8). The proportion of activated CD4^+^ T effector cells (CD4 and Granzyme B positive) and activated CD8^+^ T effector cells (CD8 and Granzyme B positive) was found to be higher among patients who responded to camrelizumab (Fig. 5b, c). As shown in Fig. 5b, the proportion of activated CD4^+^ T effector cells increased in the tumor region after ipilimumab treatment in one respond patient (P7), and decreased in the tumor region after ipilimumab treatment in one non-respond patient (P8). As shown in Fig. 5c, the proportion of activated CD8^+^ T effector cells increased in the tumor region after ipilimumab treatment in all the 4 patients. mIF assays were also conducted in four additional patients without paired tumor tissue samples before ipilimumab or before camrelizumab treatment (Fig. 5d and Supplementary Fig. 6). Similarly, the proportion of both activated CD4^+^ (Fig. 5e) and CD8^+^ T effector cells (Fig. 5f) in the tumor region was higher in patients who responded to camrelizumab than in those who did not. These are preliminary findings and more comprehensive studies with larger sample sizes are needed to definitively establish this relationship.

### Ipilimumab-induced manifested B cell infiltration in tumor tissue as well as in peripheral blood from anti-PD-1 non-responder

**Fig. 6:**
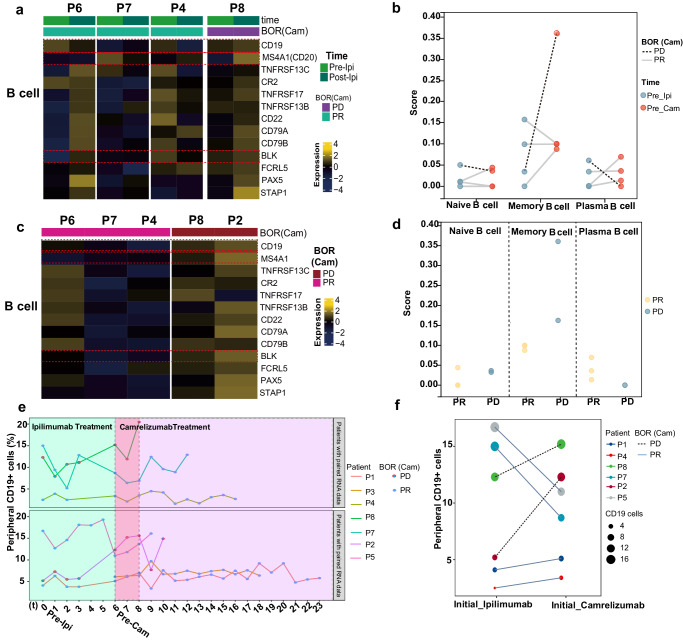
Different distribution of B cell subtypes in tumor tissues and peripheral blood of nasopharyngeal carcinoma patients with different responses to ICI therapy. **a** Heatmap showing the expression of B lineage associated genes in representative patients pre-ipilimumab and post-ipilimumab treatment by CIBERSORT. **b** Three kinds of tumor infiltrating B cells, naive B cell, memory B cell, and plasma B cell in representative patients were plotted according to CIBERSORT. **c** Heatmap showing the expression of B lineage associated genes in representative patients post-camrelizumab treatment by CIBERSORT. **d** Three kinds of tumor infiltrating B cells, naive B cell, memory B cell, and plasma B cell in representative patients post-camrelizumab treatment. **e** Percentage of peripheral CD19^+^ cells detected by flow cytometry was performed in patients at different treatment time points. **f** Different change trends of peripheral CD19^+^ cells before and after ipilimumab treatment by flow cytometry.

A trend of more B cell infiltration can be observed in the NPC TME of non-responders after ipilimumab treatment. (Fig. 4). B cell subtype analysis in patients with paired samples provided further insights into the alteration of infiltrated B cell populations. Expression of B cell-related markers CD19, CD20, and BLK was decreased in response patients (P6, P7, and P4) but increased in non-response patients (P8) after ipilimumab treatment (Fig. 6a). B cell subtypes were estimated by CIBERSORT for each patient. After ipilimumab treatment, as for response patients, P6 had intrinsically higher plasma B cell and elevated naive, memory B cell; P4 had elevated plasma and exhausted naïve, while no change was found in memory B cell; P7 had elevated plasma B cell, while no obvious change was found in naïve and memory B cell. However, non-response patient (P8) had an obviously enriched memory B cell and exhausted plasma B cell (Fig. 6b). After ipilimumab treatment, the expression of B cell-related genes (*CD19*, *MS4A1*, and *BLK*) were highly expressed in non-response patients (Fig. 6c). Higher frequency of naïve and memory B cell and lower level of plasma cell can be found in tumor tissue of non-responders compared with those of responders (Fig. 6d).

Notably, analysis of B cell subtypes in peripheral blood before and during sequential ICIs treatment was concordant with the findings in tumor samples (Fig. 6e). Comparing the data pre- or post-ipilimumab treatment, the percentage of circulating CD19^+^ B cell in the response group decreased or slightly increased, but the percentage of this B cell subtype in the non-response group increased obviously (Fig. 6f). We also observed changes in other circulating immune cells, including CD4^+^, CD8^+^ T cells and NK cells. In general, though dynamics of these peripheral circulating immunes could be observed along with CD19^+^ B cells, patterns of dynamic changes in each patient were highly heterogeneous and not associated with responses to Camrelizumab. (Supplementary Fig. 7).

## Discussion

Here, sequential ipilimumab and anti-PD-1 immunotherapy were investigated to treat patients with advanced relapsed NPC based on 2 phase I study. We first observed a favorable efficacy in these sequential treatment settings. Furthermore, potential mechanisms underlying the response to treatment was explored. Ipilimumab putatively induced tumor and immune microenvironment remodeling contributing to the response of sequential anti-PD-1 treatment. Bulk RNA sequencing of biopsy samples showed that TME was remodeled from immune-excluded/desert to immune-inflamed tumor in responders, including enriched pro-tumor immunity signatures and anti-tumor cytokines, increased density of effector T-cell in tumor-infiltrating lymphocytes (TILs), and elevated PD-L1 expression. While enriched naive memory B cell and plasma B cell deficiency were observed in non-responders.

In recent years, multiple anti-PD-1 antibodies have shown clinical efficacies in prior chemo-failed R/M NPC patients, with single-agent efficiencies of 20% to 30%^6,7,9,10^. The limitations of PD-1/PD-L1 monotherapy efficacy have ushered in the explosion of dual immunotherapy. Among them, the combination of PD-1 antibody nivolumab + CTLA4 antibody ipilimumab has been granted several FDA indications as of now. In a single arm phase II study of combination therapy in RM-NPC, there is 38% objective response rate^11^. In this study, we explored this sequential approach by connection of 2 phase I trials for the first time and observed a favorable efficacy compared with existing data. The proportion of PR patients was higher than the 20–30% reported in the previous studies using anti-PD-1 monotherapy and ~38% of anti-CTLA-4 and anti-PD-L1 combination therapy.

Although combination therapy strategies have been approved in fields such as melanoma, toxicity issues remain a significant consideration in clinical practice. Combination therapy is associated with increased toxicity, often requiring dose adjustments. For instance, the approved first-line treatment dose for advanced melanoma combines 1 mg/kg of nivolumab with 3 mg/kg of ipilimumab. Nevertheless, this combination approach has a relatively high adverse event rate, with a 55% incidence rate of grade 3–4 adverse events related to treatment observed in the CheckMate 067 study^20^. Even when the ipilimumab dose was reduced to 1 mg/kg in the CheckMate 511 study, 34% of patients still experienced treatment-related grade 3–4 adverse events. The impact of this dose adjustment on efficacy remains to be assessed during follow-up^21^. Importantly, in principle, CTLA-4 mediates the suppression of T cell activation in the antigen-presentation phase, while PD-1 mediates immune suppression in the antigen-elimination phase within the tumor^22^. The CTLA-4 inhibitor primarily functions during the immune escape initiation phase and does not require continuous use. This temporal characteristic suggests that sequential therapy, administering anti-CTLA4 followed by anti-PD-1 treatment, could potentially synergize the effects of these two drugs, making the toxicity more manageable. Previous trials in melanoma support this, showing that sequential therapy was associated with better survival outcomes than monotherapy and dual immunotherapy^13^. Therefore, sequential therapy may offer a more efficacious and safer alternative for NPC patients.

Previous studies revealed the heterogeneity of immune cell infiltrations in treatment-naive NPC patients^23,24^. Especially, intra-tumoral T cell infiltration largely impacts the efficacy of immunotherapy^25,26^. Furthermore, a high density of TILs was associated with favorable survival outcomes in NPC patients^27,28^. Previous studies have shown that anti-CTLA-4 treatment depleted intertumoral Treg cells via antibody-dependent cellular cytotoxicity, followed by anti-PD-1 significantly elevated the frequency of granzyme B + CD8+ and CD4+ cytotoxic T lymphocytes in mouse model^12,29^. In metastatic melanoma, all PD-1 blockade responders showed an increase in TCR clonality due to prior CTLA-4 blockade, however, the effect of anti-CTLA-4 on TME has not been further analyzed^18^. In the current study, intra-tumoral accumulation of CD4^+^/CD8^+^ T cells after ipilimumab treatment were found in samples with remarkable clinical benefit from sequential camrelizumab treatment. These increased intra-tumoral CD4^+^/CD8^+^ cells might be the final effectors contributing to tumor eradication under the sequential activation from anti-PD-1 blockade. Thus, prior ipilimumab induction may trigger intra-tumoral recruitment of CD4^+^/CD8^+^ T cells from peripheral blood or tumor parenchyma switching tumor microenvironment from immune-excluded/desert to immune-inflamed status, so called from “cold” to “hot” status. TME is critical for tumor development, invasion and metastasis, and its immune-inflamed state significantly affects the antitumor effects of drugs. Many investigators have demonstrated that better antitumor outcomes can be achieved by targeting therapeutic strategies that remodeling the tumor microenvironment^30–32^. Our Bulk RNA sequencing of biopsy samples confirmed such inflamed TME changes that anti-tumor immunity signatures and some pro-tumor immunity signatures were enriched, while tumor stroma, malignant cells, and tumor angiogenesis related genes were downregulated after ipilimumab treatment. But further studies are needed to clearly elucidate the mechanisms by which ipilimumab treatment leads to T-cell infiltration into the TME.

Tumor-infiltrating B cells have been detected in various solid tumors, and tumor progression is influenced by the interaction of B cells and T follicular helper cells^33^. However, the direct role of B cells in modulating the efficacy of cancer therapy remains controversial. Previous reports found that B cells were associated with prolonged survival, and showed a dual effect on recurrence and tumor progression^34^. On the one hand, they negatively regulate tumor activity through secreting immunoglobulins (Igs) to promote the T cell response and directly kill cancer cells, and on the other hand, they positively regulate tumor activity by producing immunosuppressive cytokines^35^. Thus, B cells in NPC-associated TME were analyzed in this study. In the present study, after ipilimumab induction, expression of B cell lineage marker genes such as *CD19*, *CD20*, and *BLK* was decreased in response patients but increased in non-response patient. Similar trends in B cell changes were also observed in peripheral blood. In parallel, a higher frequency of naive and memory B cell in tumor tissue of non-response patients was also observed. Nevertheless, it was interesting to find a depleted plasma cell signature in non-response patients. In line with our findings, recent data showed that tumor-infiltrating B cells, especially plasma cells can support antitumor immune responses^36^. In non-small cell lung cancer intratumoral plasma cell subsets can be used to predict the efficacy of atezolizumab^37^. Similar results were found in renal cell cancer, where intratumoral B cell maturation and antibody production was associated with response to immunotherapy^38^. Combining our results and existing evidence suggests that TME contains a heterogeneous population of B cells with functionally distinct subsets, contributing to both pro- as well as anti-immune responses. The various TME may determine whether B cells serve a pro- or an antitumorigenic function. However, this study did not directly evaluate the proportion of B cells in tumor tissue, thus further studies, particularly in prospective trials, are needed to validate our results from the sequencing analysis. In general, TME was remodeled from immune-excluded/desert to immune- inflamed status in camrelizumab-response patients. While, the presence of B cell signature, especially naive and memory B cell were associated with insensitivity of camrelizumab treatment. Therefore, anti-B-cell molecules or putative agents promoting plasma cell differentiation may serve the optimal strategies for patients who were resistant to anti-PD-1 treatment in the future.

Some researchers found that PD-L1 overexpression is common in NPC patients^39^. And clinical trials, such as KEYNOTE-028 proved that anti PD-1 antibody had antitumor activity and a manageable safety profile in RM-NPC patients with PD-L1-positive^7^. In addition, PD-L2 is one of the important ligands in the PD-1 signaling pathway. It encodes a protein that can bind to PD-1, thereby exerting the effect of inhibiting the function of immune cells. Previous studies have suggested that combined analysis of PD-L1 and PD-L2 can better predict the efficacy of immunotherapy^40,41^. In this study, we found that higher PD-L1/PD-L2 expression at baseline or up-regulation after ipilimumab were associated with responding to anti-PD-1 treatment. Meanwhile, the up-regulation of PD-L1 was found mainly in tumor cells by multiple immunofluorescences. Therefore, we consider that ipilimumab upregulated PD-L1 or PD-L2 on the surface of tumor cells, setting the conditions for subsequent anti-PD-1 treatment^42^. In addition, we also found that in non-response patients, although PD-L1 was not up-regulated, other immune checkpoints, such as *TIGIT* and *BTLA*^43–46^, had a high level of expression. At present, relevant pre-clinical studies and clinical trials for these checkpoints are underway.

The study was limited by a small sample size and the majority of the results were based on retrospective observational data. We were unable to establish control groups such as single-drug and concurrent treatment. The possibility of selection bias exists because not all patients who received ipilimumab also received camrelizumab. Therefore, more in-depth studies are needed to reveal the heterogeneity of the NPC TME, and to explore the factors that influence the response to immunotherapy.

In conclusion, the current study demonstrated that after failure of first-line platinum-based chemotherapy and second-line chemotherapy, some patients with advanced NPC may benefit from sequential anti-CTLA4 and anti-PD-1 immunotherapy. Expression profile analysis suggested that TME remodeling in these patients after ipilimumab treatment. For non-response patients, our results have also provided novel insights that novel therapeutic strategies should be developed to target naive/memory B cell or promote plasma cell differentiation.

## Methods

### Patient samples and treatment strategy

The ipilimumab phase I trial^19^ (NCT02516527) enrolled patients with advanced solid tumor who failed on at least two lines of systemic treatment (including platinum-based doublet chemotherapy), ECOG PS of 0–1, adequate organ function, and without CNS metastases, autoimmune disease. Eligible patients received 3 or 10 mg/kg ipilimumab treatment every 3 weeks up to 4 cycles or progressive disease (PD), then enter maintenance phase, every 12 weeks, starting at week 24 until PD. We consecutively collected the treatment information of those NPC patients in this ipilimumab phase I trial and received subsequential anti PD-1 treatment trial (NCT02721589). The washout period should be 4 weeks or longer. Tumor samples prior ipilimumab or prior anti PD-1 or at resistance were obtained for RNA-seq, and matched peripheral blood was collected (Fig. 1). Written informed consent was obtained from all participants. Ethical committee approval was obtained from the Institutional Review Board of Sun Yat-sen University Cancer Center (SL-B2021-445-01), in accordance with the Declaration of Helsinki. This study is compliant with the ‘Guidance of the Ministry of Science and Technology (MOST) for the Review and Approval of Human Genetic Resources’, which requires formal approval for the export of human genetic material or data from China (Application acceptance Number: 2022SLGH2233; 2022SLCJ1127).

### RNA-seq and data analysis

Total RNA was isolated from FFPE samples using AmoyDx® FFPE RNA Extraction Kit. For RNA-seq, cDNA libraries were generated using a TruSeq RNA Sample Preparation kit (Illumina) according to the manufacturer’s protocol, and sequenced on ILLUMINA NOVASEQ 6000 (Illumina Inc., CA, USA). Paired-end reads were then mapped to the Homo sapiens genome assembly GRCh37 (hg19) using STAR32 (version 020201) with transcriptome annotation (Genecode version 20). The expression levels of genes were quantified by Transcript per Million.

### Immunofluorescence analysis

Multiplex staining was performed using the PANO 7-plex IHC kit, catalog 0004100100 (Panovue, Beijing, China). According to the manufacturer’s instructions, samples were incubated with primary antibodies, while HRP-labeled secondary antibodies were incubated and tyrosine signal amplification (TSA) was performed to label the antigens. After each TSA, primary and secondary antibodies were removed using microwave thermal repair, and samples were eluted before the next antigen was labeled. After all antigen labeling was completed, the nuclei were labeled with 4′-6′-diamidino-2-phenylindole (DAPI, SIGMA-ALDRICH). The labeled samples were scanned using the Mantra System (PerkinElmer, Waltham, Massachusetts, US). For each case, a representative region of interest was selected by the pathologist and 12–20 fields of view were acquired as multispectral images at 20× resolution. The multispectral images were analyzed by InForm cell analysis software (Version 2.4, PerkinElmer, Waltham, Massachusetts, US) and quantified into data. Image analysis included tissue resolution, cell segmentation, and cell quantification. Meanwhile, mIF images were quantified using Visiopharm software (Visiopharm A/S, Hørsholm, Denmark). The quantitative data were collected by R script (version 4.1.2), and the basic data such as positive cell number, positive rate and density were obtained for follow-up data analysis.

The antibodies used for staining in this study included: CD86 (Cell Signaling, CST91882, dilution 1/100), CD206 (Abcam, AB64693, dilution 1/5000), CD11B (Cell Signaling, CST49420, dilution 1/100), PD-L1 (Cell Signaling, CST13684, dilution 1/2000), FOXP3 (Biolegend, BLG320202, dilution 1/50), Granzyme B (Abcam, AB4059, dilution 1/2000), CD4 (Biolynx, BX22300130, dilution 1/200), CD8A (Cell Signaling, CST70306, dilution 1/200), PANCK (Cell Signaling, CST4545, dilution 1/200)^47^.

### Flow cytometry

Different immune cell subsets in peripheral blood were performed using flow cytometry. The following monoclonal antibodies were used: cells were stained with

anti-CD3, anti-CD19, anti-CD56 antibodies, anti-CD4, anti-CD16, anti-CD25, and anti-CD8. All antibodies were purchased from BD Biosciences and diluted according to manufacturer instructions (tube 1, 1:200 dilution; tube 2, 1:2.5). T cells were identified as CD3^+^ and then divided into CD4^+^ and CD8^+^ populations. CD25 expression was determined on CD4^+^ T cells. The samples were run on a BD FACSCalibur (*BD* Biosciences) and analyzed using BD Cellquest 5.2.1 software. The gating strategies used for cell sorting can be found in Supplementary Fig. 8^48^.

### Quantification of EBV viral loads

The EBV-DNA load in plasma was detected by real-time quantitative polymerase chain reaction. DNA was extracted using EBV-encoded RNA ISH kit (OriGene Technologies, Inc., Beijing, China), according to the manufacturer’s protocol.

### Evaluation of infiltrating immune cells in the TME

Single-sample gene set enrichment analysis (ssGSEA) algorithm was used to evaluate the relative abundance of infiltration immune cells in the TME of NPC. The marker gene set for TME infiltration immune cell type was obtained from Bindea et al.^34^. The enrichment scores calculated by ssGSEA were used to represent the relative abundance of each TME infiltrating cell in NPC. The composition of infiltrated immune cells was evaluated by commonly used deconvolution tools, CIBERSORT via online tools (http://timer.comp-genomics.org/). To visualize the integrated analysis of genomic alterations with gene expression patterns in TME and malignant cells before ipilimumab and camrelizumab treatment, planetary schema termed Molecular-Functional portrait (MF Portrait) of the tumor created by Bagaev et al.^49^ was generated in this study. The gene expression scores of 29 immuno-related signatures were displayed in a heatmap.

### Statistical analysis

All statistical analyses were performed using GraphPad Prism 9 and R (v4.0.5) (http://www.r-project.org). Wilcoxon test was used to test the statistical significance between two independent groups or paired groups. Pair-wise *P* < 0.05 was considered to indicate statistical significance.

### Reporting summary

Further information on research design is available in the Nature Research Reporting Summary linked to this article.

### Supplementary information

Supplementary Information

Reporting Summary

## Data Availability

The raw sequencing data generated in this study have been deposited in the GSA-Human (Genome Sequence Archive for Human in BIG Data Center, Beijing Institute of Genomics, Chinese Academy of Sciences, https://ngdc.cncb.ac.cn/gsa-human/) under the accession code HRA004417. The data are available under controlled access. Any additional information required to reanalyze the data reported in this paper is available from the lead contact upon request.

## Code availability

This paper does not report the original code.
