# Supplementary material for: Remodeling the tumor-immune microenvironment by anti-CTLA4 blockade enhanced subsequent anti-PD-1 efficacy in advanced nasopharyngeal carcinoma
Source: NPJ Precis Oncol. 2024 Mar 6;8:65. doi: 10.1038/s41698-024-00558-1 (PMC10917783; doi:10.1038/s41698-024-00558-1)
Supplement: Supplementary file 1 — Supplementary Information [file 41698_2024_558_MOESM1_ESM.pdf]

Remodeling the tumor-immune microenvironment by anti-CTLA4 blockade enhanced subsequent anti-PD-1 efficacy in advanced nasopharyngeal carcinoma

Supplementary Materials

Supplementary Table 1 Pathologic and clinical features of NPC.

Sample	Age	Gender	ECOG	Smoking History*	Differentiation	Keratinizing	WHO	TNM	Stage	Prior lines	Distant metastasis sites	Liver	LDH(U/L) B1/B2	Dose of Ipilimumab	Best Overall Response	Baseline B1/B2/ resistance (FFPE)	Number of Plasma EBV detections	RNA-Seq	mIF
P1	48	Male	0	240	Yes	No	2	T3N1M0	IV	3	2	1	338.2/484.5	10mg/kg	PR	B1	15	Yes	Yes
P2	32	Male	1	0	No	No	1	cT4N2M0	IVa	6	1	0	203.3/213.0	3mg/kg	PD	B1/resistance	16	Yes	Yes
P3	69	Female	0	0	No	No	1	cT4N0M0	IVa	2	4	1	129.4/142.0	3mg/kg	PR	B1	12	Yes	Yes
P4	56	Male	0	20/day* ≥ 10 years	No	No	1	T3N3M0	IV	5	2	1	235.8/280.8	3mg/kg	PR	B1/B2	16	Yes	Yes
P5	36	Male	0	0	No	No	1	rT4N2M1	IV	2	2	1	138.0/272.1	10mg/kg	PR	B1	15	Yes	Yes
P6	29	Male	0	0	No	No	1	cT4N2M0	IV	3	1	0	273.3/262.7	10mg/kg	PR	B1/B2	19	Yes	Yes
P7	57	Male	1	0	No	No	1	rT0N1M1	IV	2	1	1	182.0/220.8	10mg/kg	PR	B1/B2	17	Yes	Yes
P8	23	Female	1	0	No	No	1	T4N2M0	IVa	3	3	1	367.5/601.4	10mg/kg	PD	B1/B2	15	Yes	Yes

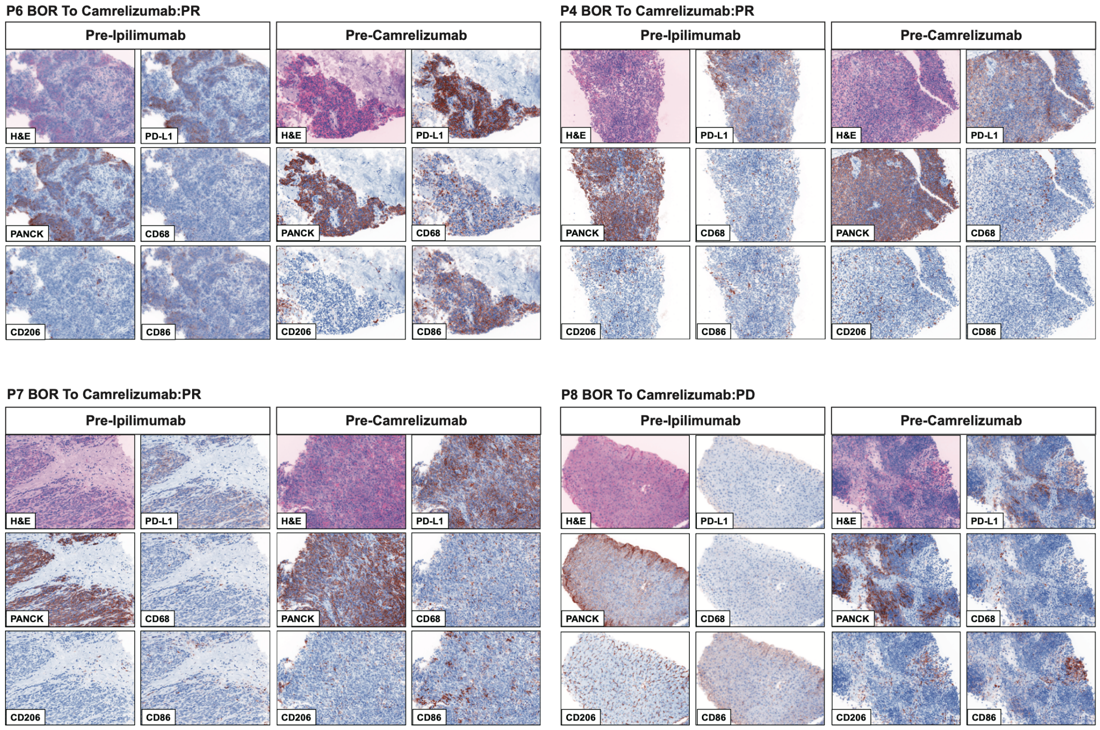

Supplementary Fig. 1 Ipilimumab treatment induced CD4+ and CD8+ T cells activation and tumor accumulation. Representative quantitative image analysis using the Visiopharm software of FFPE samples obtained from patients P6, P7, P4, and P8 pre-and post-ipilimumab treatment.

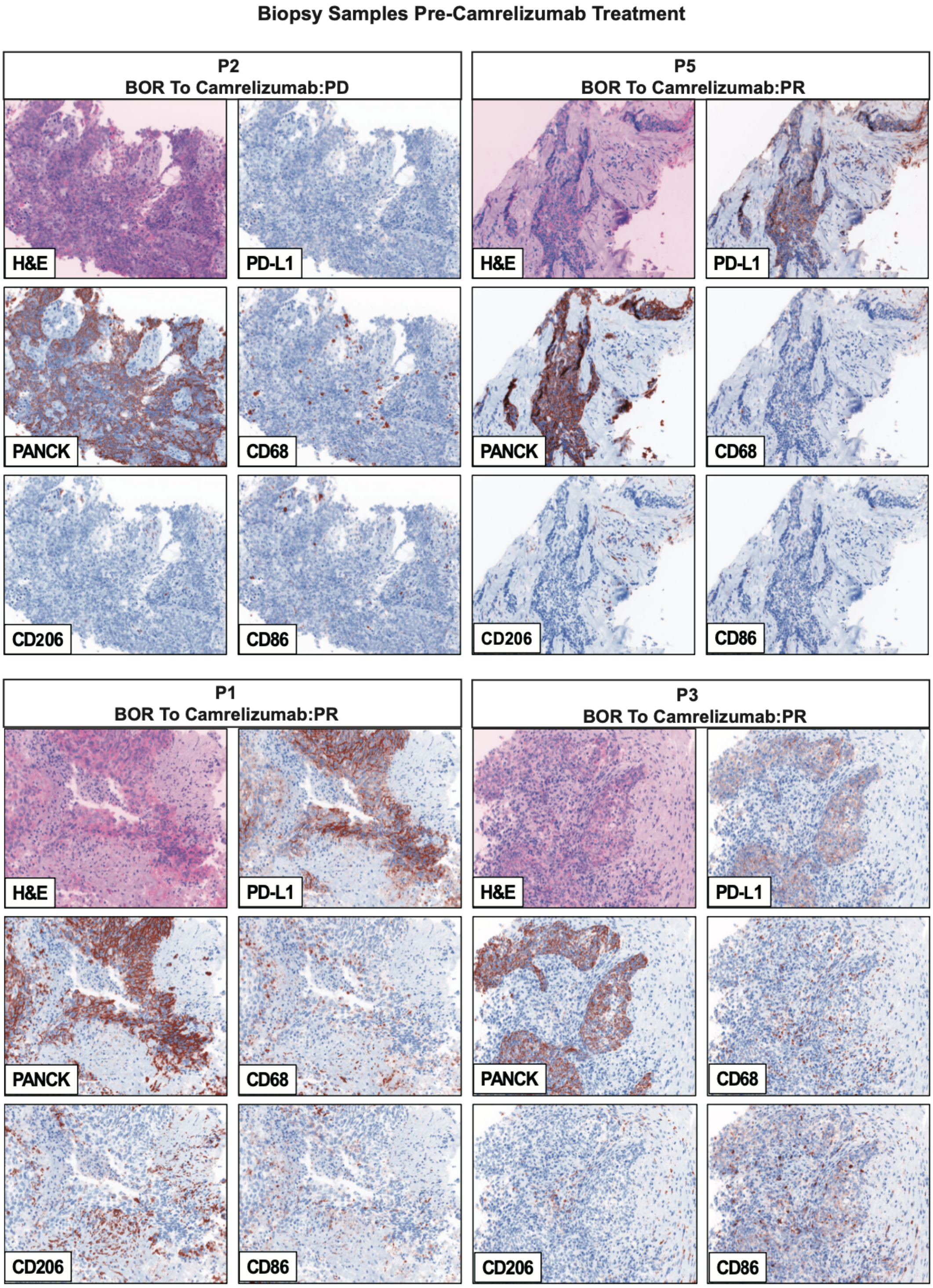

Supplementary Fig. 2 Ipilimumab treatment induced CD4+ and CD8+ T cells activation and tumor accumulation. Representative quantitative image analysis using the Visiopharm software of FFPE samples obtained from patients P2, P5, P1, and P3 before Camralizumab treatment.

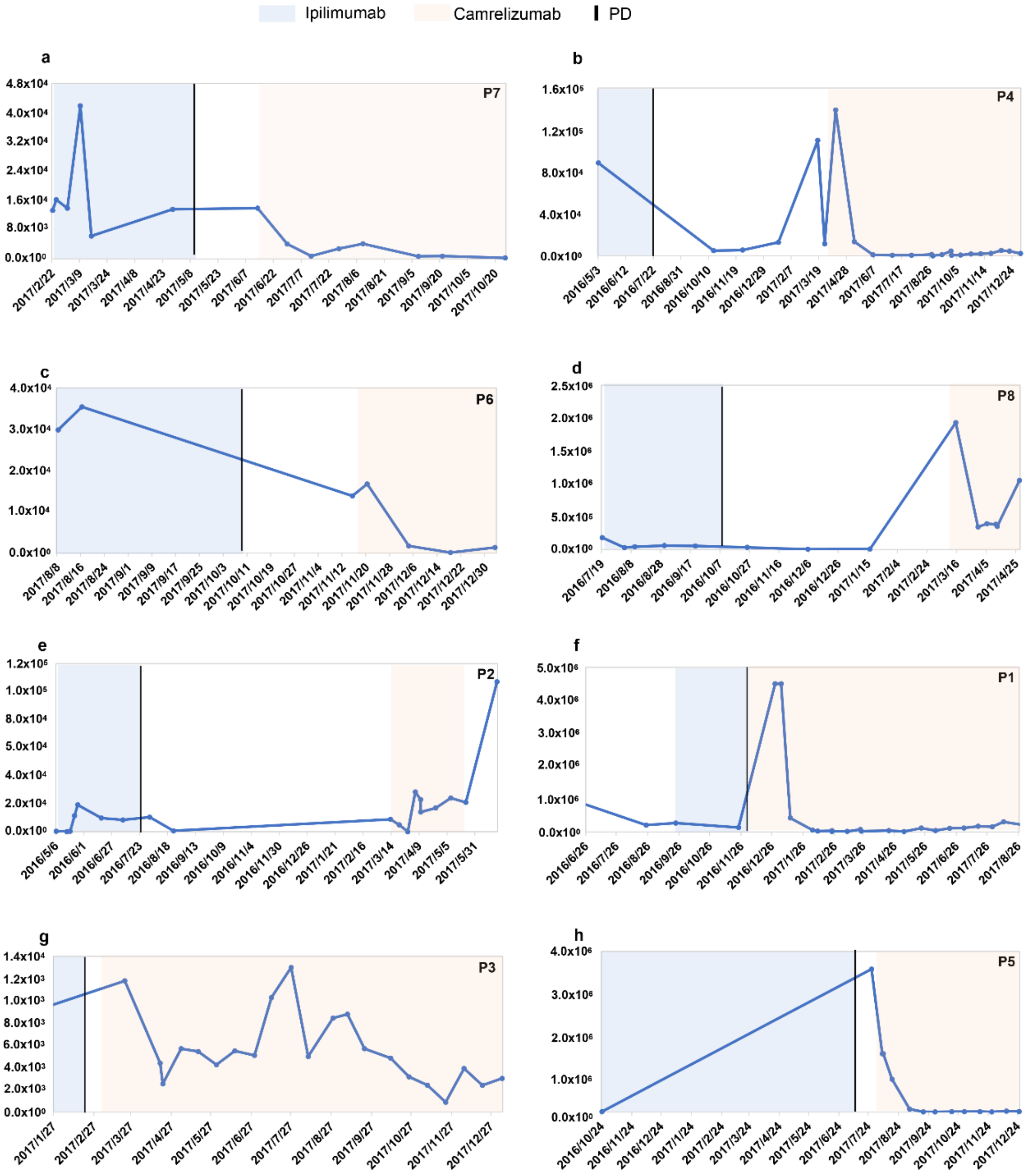

Supplementary Fig. 3 Longitudinal follow-up of serum EBV DNA in NPC patients. (a) Serum EBV DNA level from P7. (b) Serum EBV DNA level from P4. (c) Serum EBV DNA level from P6. (d) Serum EBV DNA level from P8. (e) Serum EBV DNA level from P2. (f, g, h) Serum EBV DNA level from P1, P3 and P5.

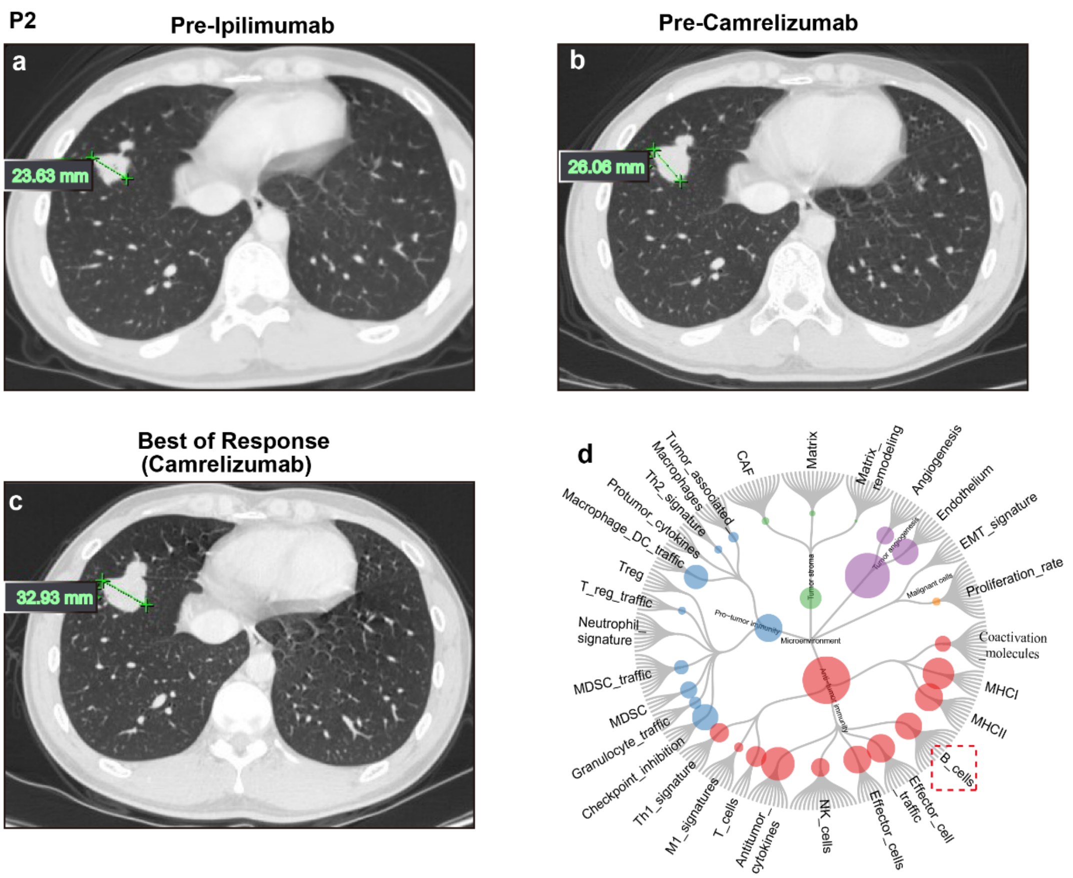

Supplementary Fig. 4 CT images and tumor microenvironment of patients in non-response group. (a-c) CT images of pre-ipilimumab, pre-camrelizumab and post-camrelizumab for P2. (d) Molecular Functional Portrait of pre-camrelizumab for P2.

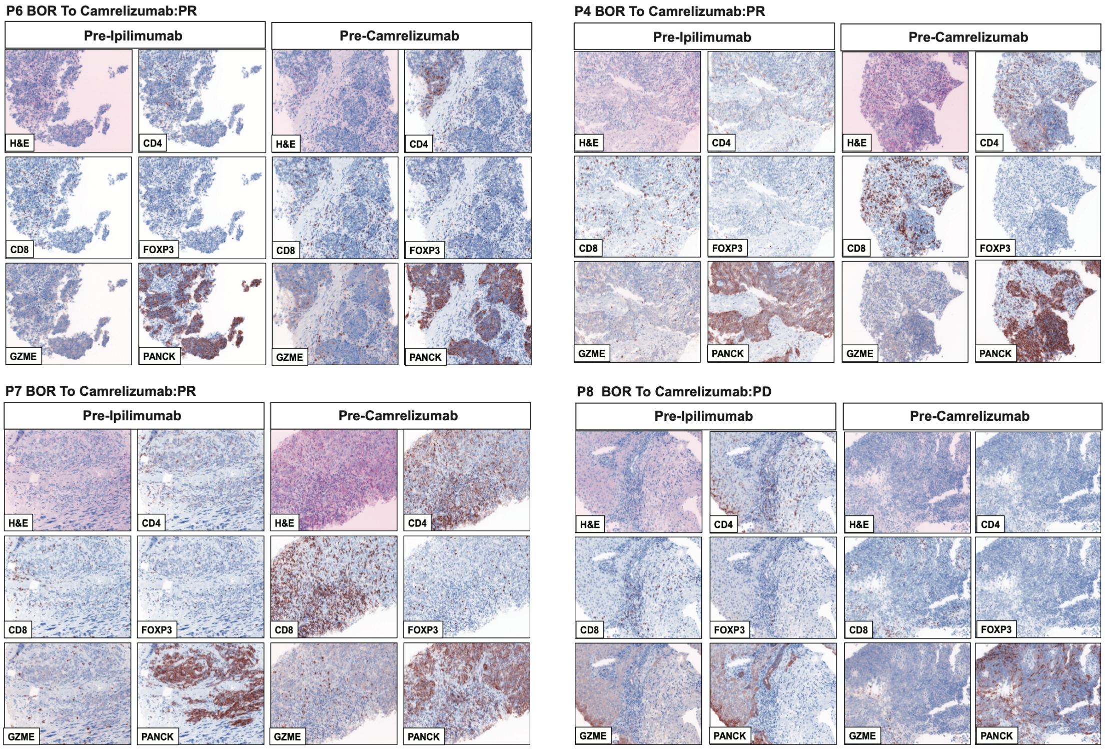

Supplementary Fig. 5 PD-L1 and other immune checkpoint molecular expression in TME. Representative quantitative image analysis using the Visiopharm software of FFPE samples obtained from patients P6, P7, P4, and P8 pre-and post-ipilimumab treatment.

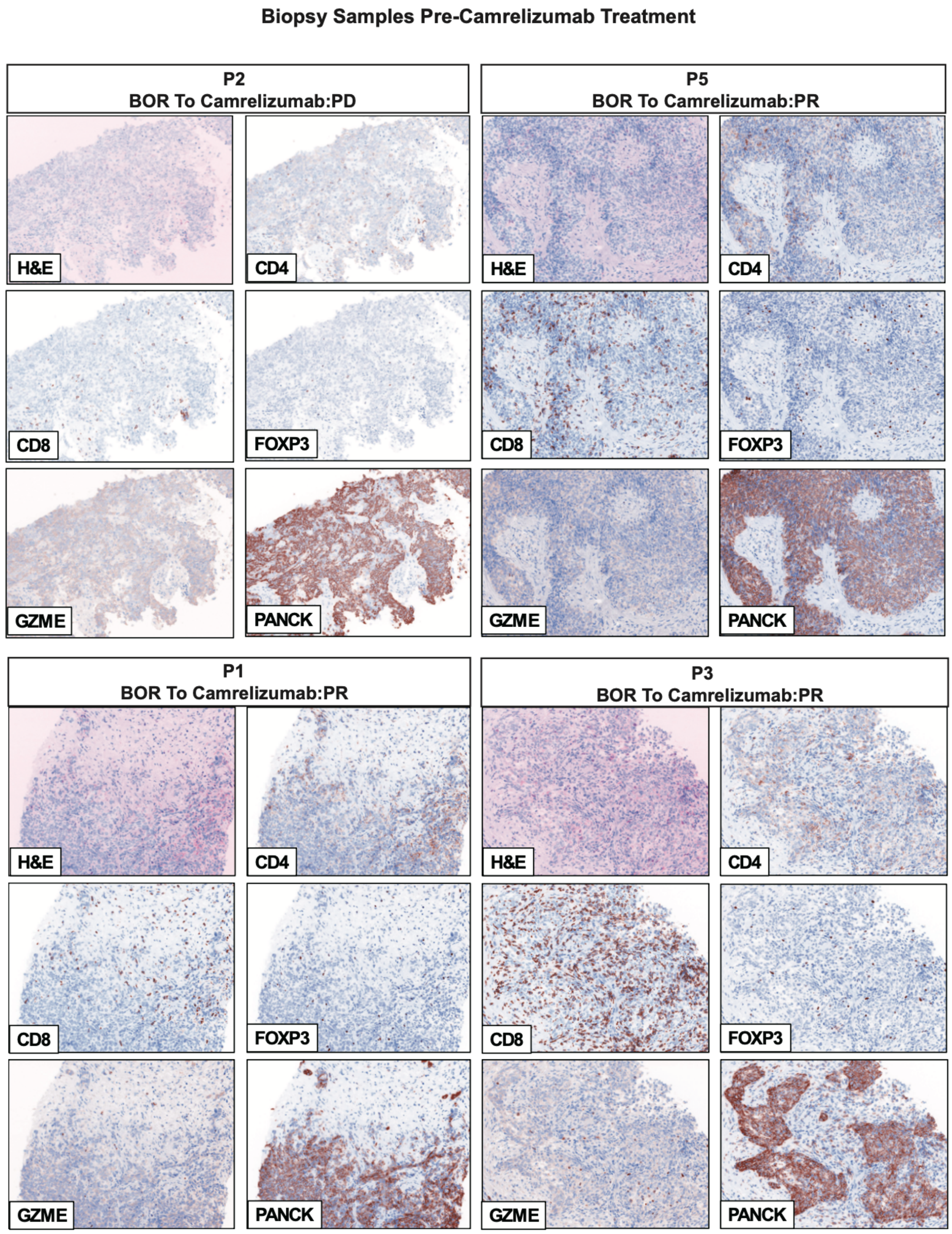

Supplementary Fig. 6 PD-L1 and other immune checkpoint molecular expression in TME. Representative quantitative image analysis using the Visiopharm software of FFPE samples obtained from patients P2, P5, P1, and P3 before Camrelizumab treatment.

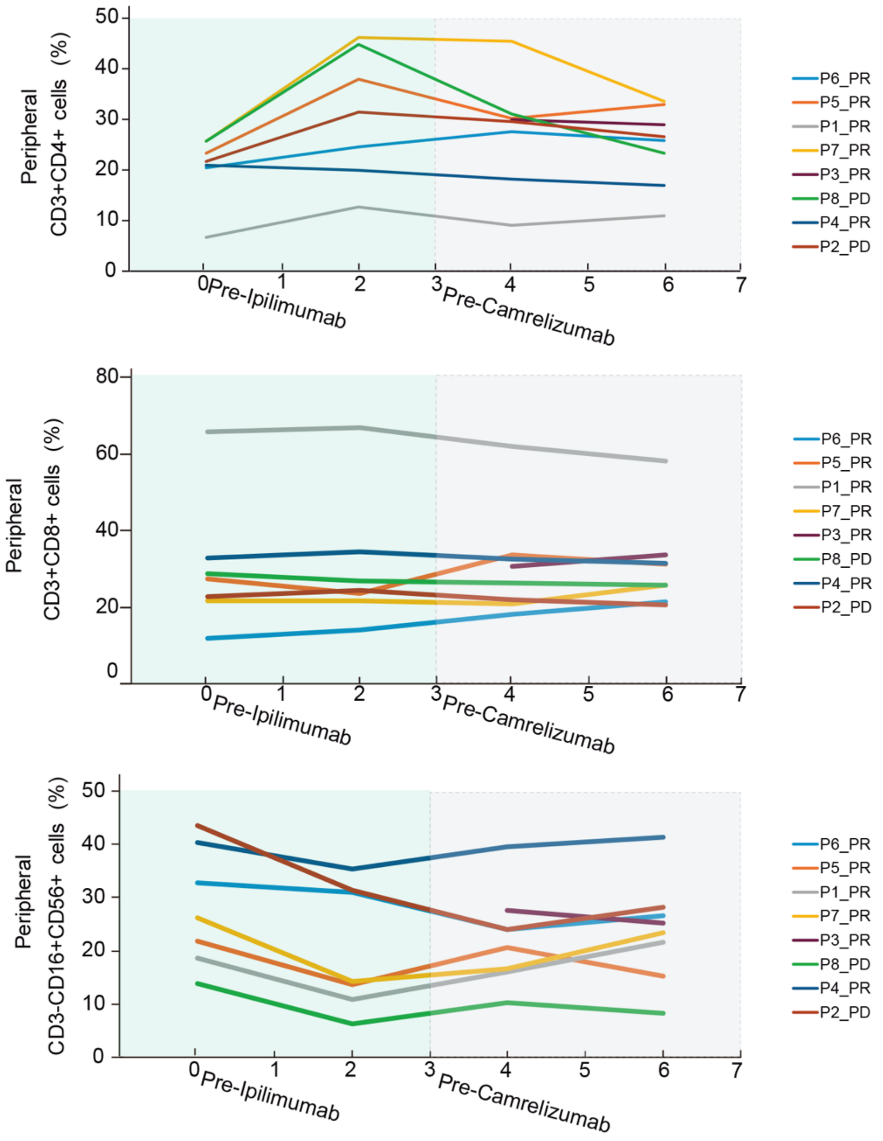

Supplementary Fig. 7. Peripheral CD3+CD4+ cells, CD3+CD8+ cells, and CD3-CD16+CD56+ cells detected by flow cytometry was performed in patients at different treatment time points.

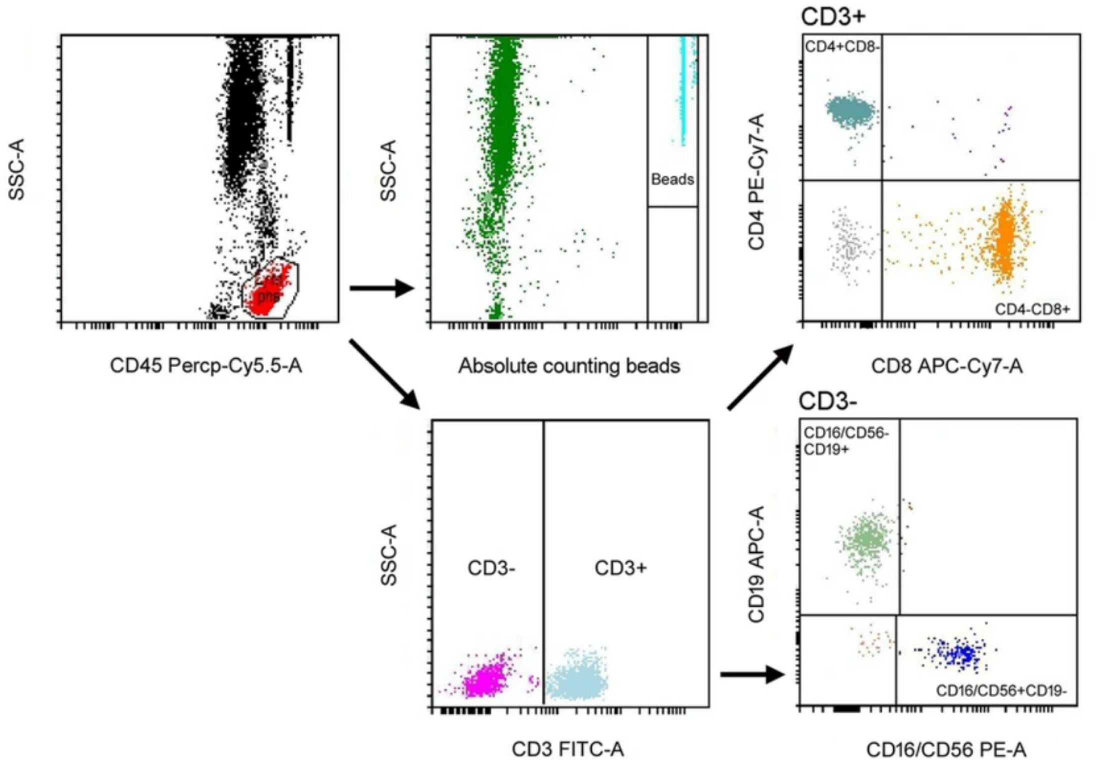

Supplementary Fig. 8. Gating strategies of lymphocyte parameter analysis. (Cited to Immun Ageing. 2022;19(1):42. Fig. 2A)
