# Supplementary material for: Remodeling the tumor-immune microenvironment by anti-CTLA4 blockade enhanced subsequent anti-PD-1 efficacy in advanced nasopharyngeal carcinoma
Source: NPJ Precis Oncol. 2024 Mar 6;8:65. doi: 10.1038/s41698-024-00558-1 (PMC10917783; doi:10.1038/s41698-024-00558-1)
Supplement: Supplementary file 2 — Reporting Summary [file 41698_2024_558_MOESM2_ESM.pdf]

nature portfolio

Corresponding author(s): Hongyun Zhao, Li Zhang

Last updated by author(s): Dec 16, 2023

Reporting Summary

Nature Portfolio wishes to improve the reproducibility of the work that we publish. This form provides structure for consistency and transparency in reporting. For further information on Nature Portfolio policies, see our Editorial Policies and the Editorial Policy Checklist.

Statistics

For all statistical analyses, confirm that the following items are present in the figure legend, table legend, main text, or Methods section.

n/a	Confirmed	
☐	☒	The exact sample size (*n*) for each experimental group/condition, given as a discrete number and unit of measurement
☐	☒	A statement on whether measurements were taken from distinct samples or whether the same sample was measured repeatedly
☐	☒	The statistical test(s) used AND whether they are one- or two-sided<br>*Only common tests should be described solely by name; describe more complex techniques in the Methods section.*
☐	☒	A description of all covariates tested
☒	☐	A description of any assumptions or corrections, such as tests of normality and adjustment for multiple comparisons
☐	☒	A full description of the statistical parameters including central tendency (e.g. means) or other basic estimates (e.g. regression coefficient) AND variation (e.g. standard deviation) or associated estimates of uncertainty (e.g. confidence intervals)
☒	☐	For null hypothesis testing, the test statistic (e.g. *F*, *t*, *r*) with confidence intervals, effect sizes, degrees of freedom and *P* value noted<br>*Give P values as exact values whenever suitable.*
☒	☐	For Bayesian analysis, information on the choice of priors and Markov chain Monte Carlo settings
☒	☐	For hierarchical and complex designs, identification of the appropriate level for tests and full reporting of outcomes
☒	☐	Estimates of effect sizes (e.g. Cohen's *d*, Pearson's *r*), indicating how they were calculated

Our web collection on statistics for biologists contains articles on many of the points above.

Software and code

Policy information about availability of computer code

Data collection	No software was used.
Data analysis	All statistical analyses were performed using GraphPad Prism 9 and R (v4.0.5) (http://www.r-project.org). Wilcoxon test was used to test the statistical significance between two independent group or paired groups. Pair-wise P<0.05 was considered to indicate statistical significance.

For manuscripts utilizing custom algorithms or software that are central to the research but not yet described in published literature, software must be made available to editors and reviewers. We strongly encourage code deposition in a community repository (e.g. GitHub). See the Nature Portfolio guidelines for submitting code & software for further information.

Data

Policy information about availability of data

All manuscripts must include a data availability statement. This statement should provide the following information, where applicable:

- Accession codes, unique identifiers, or web links for publicly available datasets
- A description of any restrictions on data availability
- For clinical datasets or third party data, please ensure that the statement adheres to our policy

The raw sequencing data generated in this study have been deposited in the GSA-Human (Genome Sequence Archive for Human in BIG Data Center, Beijing Institute of Genomics, Chinese Academy of Sciences, http://gsa.big.ac.cn/gsa-human) under the accession code HRA004417. The data are available under controlled access.

Research involving human participants, their data, or biological material

Policy information about studies with human participants or human data. See also policy information about sex, gender (identity/presentation), and sexual orientation and race, ethnicity and racism.

Reporting on sex and gender	Most patients are male (n=6, 75%).
Reporting on race, ethnicity, or other socially relevant groupings	All patients are Chinese.
Population characteristics	Median age was 42 year (range 23-69 years) and most patients are male (n=6, 75%). Liver metastases are found in 6 patients. There were 5 patients with ECOG PS of 0, and 3 with ECOG 1. The average prior treatment line was 3.25.
Recruitment	The ipilimumab phase I trial19 enrolled patients with advanced solid tumor who failed on at least two lines of systemic treatment (including platinum-based doublet chemotherapy), ECOG PS of 0-1, adequate organ function, and without CNS metastases, autoimmune disease. Eligible patients received 3 or 10mg/kg ipilimumab treatment every 3 weeks up to 4 cycles or progressive disease (PD), then enter maintenance phase, every 12 weeks, starting at week 24 until PD. We consecutively collected the treatment information of those NPC patients in this ipilimumab phase I trial and received subsequential anti PD-1 treatment.
Ethics oversight	Ethical approval was obtained from the Institutional Review Board of Sun Yat-sen University Cancer Center (SL-B2021-445-01), in accordance with the Declaration of Helsinki.

Note that full information on the approval of the study protocol must also be provided in the manuscript.

Field-specific reporting

Please select the one below that is the best fit for your research. If you are not sure, read the appropriate sections before making your selection.

☒ Life sciences ☐ Behavioural & social sciences ☐ Ecological, evolutionary & environmental sciences

For a reference copy of the document with all sections, see nature.com/documents/nr-reporting-summary-flat.pdf

Life sciences study design

All studies must disclose on these points even when the disclosure is negative.

Sample size	This is a retrospective study, no sample size was calculated.
Data exclusions	No data were excluded from the analyses.
Replication	N/A
Randomization	N/A
Blinding	N/A

Reporting for specific materials, systems and methods

We require information from authors about some types of materials, experimental systems and methods used in many studies. Here, indicate whether each material, system or method listed is relevant to your study. If you are not sure if a list item applies to your research, read the appropriate section before selecting a response.

Materials & experimental systems

n/a	Involved in the study	
☐	☒	Antibodies
☒	☐	Eukaryotic cell lines
☒	☐	Palaeontology and archaeology
☒	☐	Animals and other organisms
☐	☒	Clinical data
☒	☐	Dual use research of concern
☒	☐	Plants

Methods

n/a	Involved in the study	
☒	☐	ChIP-seq
☒	☐	Flow cytometry
☒	☐	MRI-based neuroimaging

Antibodies

Antibodies used	CD86 (Cell Signaling, CST91882), CD206 (Abcam, AB64693), CD11B (Cell Signaling, CST49420), PD-L1 (Cell Signaling, CST13684), FOXP3 (Biolegend, BLG320202), Granzyme B (Abcam, AB4059), CD4 (Biolynx, BX22300130), CD8A (Cell Signaling, CST70306), PANCK (Cell Signaling, CST4545).
Validation	N/A

Clinical data

Policy information about clinical studies

All manuscripts should comply with the ICMJE guidelines for publication of clinical research and a completed CONSORT checklist must be included with all submissions.

Clinical trial registration	NCT02516527 and NCT02721589
Study protocol	Available from the lead contact upon request
Data collection	Population characteristics, treatment, survival time
Outcomes	PFS, OS, AE

Plants

Seed stocks	N/A
Novel plant genotypes	N/A
Authentication	N/A
